# Epac inhibits migration and proliferation of human prostate carcinoma cells

**DOI:** 10.1038/sj.bjc.6605439

**Published:** 2009-11-17

**Authors:** M Grandoch, A Rose, M ter Braak, V Jendrossek, H Rübben, J W Fischer, M Schmidt, A A Weber

**Affiliations:** 1 Department of Pharmacology, University of Essen Medical School, Essen, Germany; 2 Institute for Cell Biology, Department of Molecular Cell Biology, University of Duisburg-Essen, Essen, Germany; 3 Department of Urology and Urooncology, University of Essen Medical School, Essen, Germany; 4 Department of Molecular Pharmacology, University of Groningen, Groningen, The Netherlands

**Keywords:** prostate cancer, cAMP, Epac

## Abstract

**Background:**

It was recently found that cAMP mediates protein kinase A-independent effects through Epac proteins. The aim of this study was to investigate the role of Epac in migration and proliferation of prostate carcinoma cells.

**Methods:**

The effect of Epac activation was determined by [^3^H]thymidine incorporation and scratch assays in PC-3 and DU 145 cells. Furthermore, cytoskeletal integrity was analysed by phalloidin staining. The participation of intracellular Epac effectors such as mitogen-activated protein (MAP) kinases, Rap1- and Rho-GTPases was determined by immunoblotting and pull-down assay.

**Results:**

The specific Epac activator 8-pCPT-2′-*O*-Me-cAMP (8-pCPT) interfered with cytoskeletal integrity, reduced DNA synthesis, and migration. Although 8-pCPT activated Rap1, it inhibited MAP kinase signalling and RhoA activation. These findings were translated into functional effects such as inhibition of mitogenesis, cytoskeletal integrity, and migration.

**Conclusion:**

In human prostate carcinoma cells, Epac inhibits proliferative and migratory responses likely because of inhibition of MAP kinase and RhoA signalling pathways. Therefore, Epac might represent an attractive therapeutic target in the treatment of prostate cancer.

Migration and proliferation are key events in cancer progression and metastasis and the underlying molecular mechanisms of interest for possible treatment options (Abate-Shen and Shen, 2000; Hao *et al*, 2007). It has been reported that progression of prostate cancer, one of the leading causes of male cancer death, is associated with an activation of members of the Ras family, which in turn activate effectors, such as the mitogen-activated protein kinases (MAPKs). In fact, elevated levels of the subfamily of extracellular signal-regulated kinases 1 and 2 (ERK-1/2) could be observed in advanced prostate tumours (Gioeli *et al*, 1999; Price *et al*, 1999; Uzgare *et al*, 2003). In prostate carcinoma cells, cAMP analogues, such as Bt2-cAMP or dbcAMP, not only inhibited proliferation and migration but also induced cell differentiation (Bang *et al*, 1992, 1994; Picascia *et al*, 2002; Chen *et al*, 2005), thereby presenting potential new therapeutical treatment options.

For many years, these cAMP effects have been attributed solely to the activation of protein kinase A (PKA) (Taylor *et al*, 1990). However, recent studies have also shown the PKA-independent effects of cAMP, suggesting a possible involvement of other cAMP effectors, such as Epac (also known as cAMP-GEF (guanine nucleotide exchange factors)), acting as GEFs for Rap and Ras (Taylor *et al*, 1990; Bos, 2003; Holz *et al*, 2008). We and others have recently shown that Epac proteins have an important role in several cellular processes, including proliferation and apoptosis, possibly by their signalling capacity to ERK and phosphoinositide-3 kinase-dependent Akt (Fang and Olah, 2007; Grandoch *et al*, 2009). It is important that very recent studies could show an involvement of Epac in cell migration (Mei and Cheng, 2005; Yokoyama *et al*, 2008; Bastian *et al*, 2009), although the effects (inhibitory or stimulatory) were not consistent.

Thus far, the role of Epac on migration and proliferation has not been investigated in the prostate tumour cell lines PC-3 and DU 145. Therefore, we focused here on the question whether Epac modulates those cellular functions in highly aggressive prostate tumour cell lines with a high proliferative and metastasising capacity.

## Materials and methods

See also extended Materials and Methods section in Supplementary materials (available online).

### Cell culture and toxin treatment

PC-3 and DU 145 were cultured under standard conditions and serum-deprived 12 h before experiments. *Clostridium difficile* toxin B (TcdB) was added for 24 h at 10 pg ml^−1^ (DU 145) or 100 pg ml^−1^ (PC-3).

### Western blot

After cell lysis (1% (vol/vol) SDS and 10 mM Tris/HCl, pH 7.4), proteins were separated by SDS–PAGE (10–15%), blotted onto nitrocellulose, incubated with the indicated primary antibodies (1 : 1000, 4°C), and detected by the appropriate secondary antibodies.

### Mitogenesis and migration

DNA synthesis was measured using the [^3^H]thymidine incorporation assay. Cell migration was determined in the presence of 1 mM hydroxy urea after scratch wounding of monolayer cultures.

### Affinity precipitation of GTP-bound Rap1 and RhoA

Activated Rap1 or RhoA was extracted from cell lysates with glutathione *S*-transferase-tagged RalGDS (Rap1) or Rhotekin (RhoA), bound to Glutathione Sepharose beads. Immunoblotting was performed with anti-Rap1 and anti-RhoA antibodies.

### Immunofluorescence microscopy and flow cytometry

Cells were fixed (paraformaldehyde), permeablized (Triton X-100), and stained for filamentous actin using AlexaFluor 488-conjugated phalloidin. Cells were analysed microscopically and by flow cytometry.

### Statistics

Data are means±s.e.m. of *n* independent experiments. Statistical significance was determined by one-way analysis of variance followed by the Bonferroni test for multiple comparisons. *P*<0.05 was considered significant.

## Results

**Figure 1:**
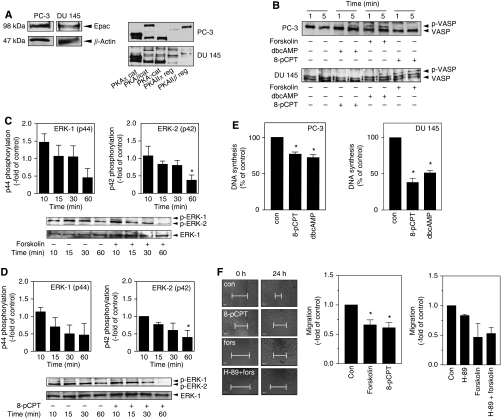
Epac expression and functional characterisation in the human prostate cancer cell lines PC-3 and DU 145. Effects of the activation of adenylyl cyclase and Epac on extracellular signal-regulated kinases 1 and 2 (ERK-1/2) phosphorylation, DNA synthesis, and migration. The effect of protein kinase A (PKA) on cell migration. (**A**) Endogenously expressed Epac and PKA (western blotting) in PC-3 and DU 145 cells. (**B**) Specificity of the Epac activator 8-pCPT-2′-*O*-Me-cAMP (8-pCPT; 300 *μ*M) as compared with the adenylyl cyclase activator forskolin (fors, 100 *μ*M) and the cAMP analogue dbcAMP (1 mM) as measured by the phosphorylation of vasodilator-activated phosphoprotein (VASP) (western blotting). (**C** and **D**) Effects of fors (100 *μ*M) and 8-pCPT (300 *μ*M) on ERK-1/2 phosphorylation in PC-3 cells. Representative immunoblots and densitometrical analyses (means±s.e.m.) from *n*=4 experiments are shown, ^*^P<0.05 *vs* control (con) (ANOVA/Bonferroni). (**E**) Effects of dbcAMP (1 mM) and 8-pCPT (300 *μ*M) on DNA synthesis ([^3^H]thymidine incorporation). Data are means±s.e.m. from *n*=4 experiments, ^*^P<0.05 *vs* control (ANOVA/Bonferroni). (**F**) Effects of fors (100 *μ*M), 8-pCPT (300 *μ*M) and H-89 (10 *μ*M) on cell migration in PC-3 cells. Microscopic analysis of the cell-free area was carried out at 0 and 24 h and the area invaded by tumour cells was calculated (migration margins are indicated). Original photographs (cell-free area indicated) and quantitative analysis (means±s.e.m.) of *n*=3 experiments are shown, ^*^P<0.05 *vs* control (ANOVA/Bonferroni). ANOVA, analysis of variance.

As shown in Figure 1A, PC-3 and DU 145 cells express Epac and the different PKA catalytic and regulatory subunits. To control the specificity of the Epac-specific activator 8-pCPT-2′-*O*-Me-cAMP (8-pCPT), we analysed the phosphorylation of vasodilator-activated phosphoprotein (VASP), which is known to be phosphorylated by PKA (Smolenski *et al*, 1998). Activation of the adenylyl cyclase by forskolin (100 *μ*M) and treatment with the cAMP analogue dbcAMP (1 mM) induced VASP phosphorylation in both cell lines through PKA. In contrast, the Epac activator 8-pCPT (300 *μ*M) did not induce VASP phosphorylation (Figure 1B).

Next we compared the effects of cAMP and 8-pCPT on the phosphorylation of ERK-1/2 in PC-3 cells. Both, forskolin and 8-pCPT induced a slow, time-dependent decrease in basal ERK-1/2 phosphorylation (Figure 1C and D). To study possible functional consequences of the Epac-mediated inhibition of the MAPK pathway, we studied the effects of Epac activation on mitogenesis in PC-3 and DU 145 cells. Both, dbcAMP (this cAMP analogue was used because of inconsistent effects of forskolin in this assay; data not shown) and 8-pCPT induced a significant decrease in DNA synthesis (Figure 1E). In addition, both forskolin and 8-pCPT significantly inhibited cell migration of PC-3 and DU 145 cells (Figure 1F). Importantly, the PKA-inhibitor H-89 (10 *μ*M) showed no significant effects on the inhibition of cell migration of PC-3 (Figure 1F) and DU 145 cells (data not shown). Furthermore, inhibition of MEK by PD98059 (10 *μ*M) and U0126 (10 *μ*M) also decreased cell migration and enhanced the inhibitory effects of forskolin and 8-pCPT (data not shown) in PC-3 cells.

**Figure 2:**
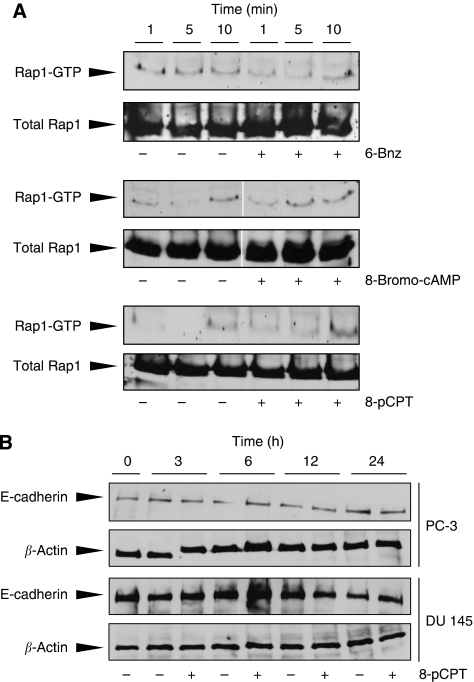
Epac-dependent effects on E-cadherin expression. Activation of Rap1 by 8-Bromo-cAMP, 6-Bnz, or 8-pCPT-2′-*O*-Me-cAMP (8-pCPT). (**A**) PC-3 and DU 145 cells were stimulated for the indicated periods of time with (+) or without (−) 300 *μ*M 8-pCPT. Expression of E-cadherin in whole-cell lysates was detected by western blotting. Equal loading was shown by using *β*-Actin antibody. (**B**) Rap1 activation induced by 8-Bromo-cAMP (1 mM), 6-Bnz (500 *μ*M), or 8-pCPT (300 *μ*M) at 1, 5, and 10 min (pull-down/western blot).

Next, effects on the small GTPase Rap1, a downstream effector of Epac, were analysed. Incubation with the PKA-specific cAMP analogue 6-Bnz (500 *μ*M) led to a slight decrease in Rap1 activation, whereas only the nonspecific 8-Bromo-cAMP (1 mM) and the Epac-activator 8-pCPT (300 *μ*M) activated the small GTPase at 5 and 10 min, respectively (Figure 2A).

E-cadherin is highly relevant for cancer invasiveness (Behrens *et al*, 1993) and has been shown to be regulated by Rap (for review see, Price *et al*, 2004; Koistra *et al*, 2007). Thus, we next studied the expression of E-cadherin in PC-3 and DU 145 cells in response to 8-pCPT (300 *μ*M) by immunoblotting (Figure 2B). However, the Epac activator did not alter E-cadherin expression in the two cell lines.

**Figure 3:**
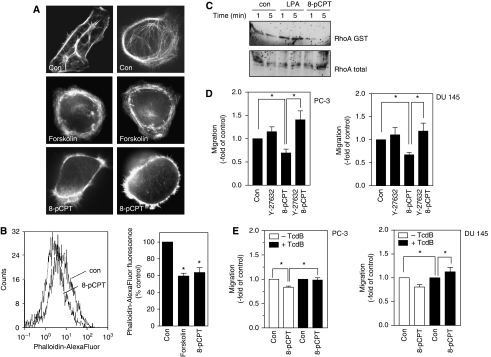
Effects of forskolin and 8-pCPT-2′-*O*-Me-cAMP (8-pCPT) on the actin cytoskeleton. (**A**) Effects of forskolin (100 *μ*M, 1 h) or 8-pCPT (300 *μ*M, 1 h) on filamentous actin (Alexa fluor 488-conjugated phalloidin staining). (**B**) Original FACS histograms and quantitative analysis of cellular Alexa fluor 488-conjugated phalloidin fluorescence. Data are means±s.e.m. from *n*=3–5 independent experiments, ^*^P<0.05 *vs* control (ANOVA/Bonferroni). (**C**) Effects of forskolin (100 *μ*M, 1 h) or 8-pCPT (300 *μ*M, 1 h) on RhoA activation (pull-down/western blot). (**D**) Effects of the Rho kinase inhibitor Y-27632 on cell migration of PC-3 and DU 145 cells. Pre-incubation with Y-27632 (10 *μ*M) for 20 min was followed by stimulation of the cells with 8-pCPT (300 *μ*M). Microscopic analysis of the cell-free area was carried out at 0 and 24 h, and the area invaded by tumour cells was calculated and quantitative analysis (means±s.e.m.) of *n*=3 experiments are shown, ^*^P<0.05 *vs* control (ANOVA/Bonferroni). (**E**) Effects of toxin B on cell migration in PC-3 and DU 145 cells. Cells were incubated for 24 h with *Clostridium difficile* toxin B (TcdB; 10 and 100 pg ml^−1^) before stimulation with the indicated agents and microscopic analysis of the cell-free area at 0 and 24 h. The area invaded by the tumour cells was calculated. Quantitative analysis (means±s.e.m.) of *n*=3 experiments are shown, ^*^P<0.05 *vs* control (ANOVA/Bonferroni). ANOVA, analysis of variance.

As cell migration is accompanied by a reorganisation of the actin cytoskeleton, we also studied the cytoskeletal actin organisation by staining F-actin with phalloidin in PC-3 cells. Although in control cells microscopically visible actin stress fibres could be detected, treatment with forskolin (100 *μ*M) or 8-pCPT (300 *μ*M) led to cell rounding accompanied by a disassembling of the stress fibres with an irregular pattern and disruption into short fragments (Figure 3A). These effects were confirmed by quantitative analysis of F-actin as measured by flow cytometry (Figure 3B). To elucidate the effect of Epac on Rho GTPases, we performed RhoA activation assays. Lysophosphatidic acid (1 *μ*M) was used as a positive control. Indeed, we could detect a decrease in RhoA activation after treatment of PC-3 cells with the Epac activator 8-pCPT (300 *μ*M; Figure 3C).

We confirmed the involvement of Rho-GTPases in the Epac-activated signalling pathways using two Rho kinase inhibitors, Y-27632 and HA-1077, respectively. Inhibition of Rho kinase by Y-27632 (10 *μ*M) reversed 8-pCPT-induced inhibition of migration in PC-3 and DU 145 cells (Figure 3D). Similar effects were also detectable after inhibition of Rho kinase by HA-1077 (10 *μ*M; data not shown). In addition, we used TcdB, a protein reported to monoglucosylate members of the Rho protein family. After pre-incubation of PC-3 and DU 145 cells for 24 h with TcdB and following stimulation with 8-pCPT, no inhibitory effects of the Epac activator could be detected (Figure 3E).

## Discussion

This study is the first to show the anti-mitogenic and anti-migratory effects of Epac activation in human prostate cancer cells.

Bang *et al* (1992) suggested that cAMP analogues might be useful in the treatment of metastatic prostate cancer. However, no discrimination between different possible cAMP effectors has been made thus far, and for a long time PKA has been assumed as the only cAMP target. Importantly, Epac proteins have been shown to act as multifunctional regulators in different cell functions, next to the canonical PKA pathway. Indeed, we were able to detect Epac as well as PKA expression in PC-3 and DU 145 cells. First, we concentrated on ERK-1/2 phosphorylation. We confirmed inhibitory effects of cAMP on the phosphorylation level of ERK-1/2, mitogenesis, and migration (Price *et al*, 1999; Carey *et al*, 2007; Hao *et al*, 2007). Interestingly, we also detected a significant inhibition of the MAPK signalling by the specific Epac activator 8-pCPT. These findings were translated into functional effects, such as an inhibition of mitogenesis and migration.

As neither forskolin nor 8-pCPT induced apoptotic effects in PC-3 cells (annexin V binding, caspase cleavage, PARP processing; data not shown), we hypothesise that the observed inhibition of cell proliferation by these compounds most likely depends on the inhibition of ERK-1/2-mediated mitogenic events.

With regard to the anti-migratory effects of Epac activation in PC-3 and DU 145 cells, our results are in line with recent findings in ES-2 ovarian carcinoma cells (Bastian *et al*, 2009). The PKA inhibitor H-89 was not able to prevent forskolin-induced inhibition of cell migration strongly pointing to an Epac-dependent signalling pathway. Interestingly, incubation of cells with the inhibitor alone led to different outcomes in PC-3 *vs* DU 145 cells. A reason for this might be the fact that H-89 is known to inhibit even more target proteins than the well-known PKA (Davies *et al*, 2000).

Furthermore, Epac-dependent Rap1 activation is shown in this study in PC-3 cells. However, no effect on E-cadherin expression, which is known to be important for cancer cell migration and tumour invasiveness (Behrens *et al*, 1993), could be detected. This is in line with recent studies also describing an Epac/Rap1-mediated inhibition of epithelial cell migration independent of a modulation of the E-cadherin expression (Lyle *et al*, 2008).

Importantly, our experiments support the conclusion that inhibition of Rho GTPases, which are crucial for cell migration (Raftopoulou and Hall, 2004) and invasion of PC-3 cells (Hodge *et al*, 2003), might cause the anti-migratory effects of Epac. Stimulation of Epac by 8-pCPT led to a complete disassembly of the F-actin cytoskeleton. Furthermore, a decrease in RhoA activation was also observed in a pull-down assay. These results are in line with the described effects of cAMP analogues on RhoA activation and do confirm the proposed cross-talk between cAMP and RhoA (Chen *et al*, 2005).

In summary, this study shows for the first time anti-proliferative and anti-migratory effects of Epac activation in human prostate cancer cells, presumably mechanistically explained by an inhibition of the MAP kinases and RhoA. As these cellular functions are crucial for tumour growth and metastasis, Epac might represent an attractive therapeutic target in the treatment of prostate cancer.
