# Supplementary material for: Epac inhibits migration and proliferation of human prostate carcinoma cells
Source: Br J Cancer. 2009 Nov 17;101(12):2038–42. doi: 10.1038/sj.bjc.6605439 (PMC2795436; doi:10.1038/sj.bjc.6605439)
Supplement: Online Supplementary Material [file 6605439x1.doc]

**Online supplementary material**

Extended ***Materials and Methods***

***Materials***- Forskolin, N^6^,2′-O-Dibutyryladenosine 3′,5′-cyclic monophosphate sodium salt (dbcAMP) and staurosporine were from Sigma-Aldrich. 6-Bnz-cAMP (B009) and 8-(4-chlorophenylthio)-2'-*O*-methyl cyclic AMP (8-pCPT-2’-*O*-Me-cAMP) (C 041) were from Biolog Life Science Institute (Bremen, Germany). PD98059, U0126, 8-bromo cyclic AMP (8-Bromo-cAMP), H-89, Y-27632 and HA 1077 were from Calbiochem-Merck Biosciences. The antibodies against ERK1 (C-16) and Rap1 (121, sc-65) were from Santa Cruz. The anti-phospho-ERK1/2 (P-ERK1/2) (9101, Thr202/Tyr204), anti-vasodilator-stimulated phosphoprotein (VASP) (3112), anti-caspase-3 (9662), anti-E-cadherin (4065) and anti-poly ADP-ribose polymerase (PARP) (9542) were from Cell Signaling Technology. The antibodies against Epac 1 (A-5, Santa Cruz Biotechnology, sc-28366), PKAα cat (C-20, Santa Cruz Biotechnology, sc-903), PKAβ cat (C-20, Santa Cruz Biotechnology, sc-904), PKAγ cat (C-20, Santa Cruz Biotechnology, sc-905), PKA Iβ reg (C-19, Santa Cruz Biotechnology, sc-907) PKA IIα reg (C-20, Santa Cruz Biotechnology, sc-908) were from Santa Cruz. The antibody against beta Actin (beta Actin antibody-Loading control, ab8227) was from Abcam. Alexa fluor 488-conjugated phalloidin and annexin V were from Invitrogene). [^3^H]thymidine (5-6 mCi/mmol) was from PerkinElmer Life Sciences.

***Cell culture -*** The human androgen-independent prostate adenocarcinoma cell line PC-3 (ATCC number CRL-1435) and the human prostate carcinoma cell line DU 145 (ATCC number HTB-81) were cultured in RPMI 1640 medium supplemented with 10% fetal calf serum and 1% penicillin/streptomycin (Invitrogen) at 37°C in a humidified 5% CO_2_ atmosphere. For the experiments, cells were seeded at a concentration of 1 x 10^6^ /ml and serum-deprived overnight. Subsequently, cells were incubated for the indicated periods of time at 37°C with the indicated agents and lysed in a buffer containing 1% (v/v) SDS and 10 mM Tris/HCl, pH 7.4, and 5 passages through a 25-gauge needle.

***Western blot***- After cell lysis, protein concentrations were determined and 20 µg of protein was incubated in Laemmli buffer for 10 min at 95°C followed by separation by SDS-PAGE on 10 - 15% acrylamide gels. After transfer to nitrocellulose membranes and an overnight incubation with the antibodies (1:1000, 4°C), the proteins were visualized by enhanced chemiluminescence (PerkinElmer Lifesciences). Quantification was performed by scanning the film with a grey scale-calibrated scanning system (HP Scanjet G4050; Phoretix Power Scan V 2003) and band intensity was calculated with LabImage 1D V 4.0 (Kapelan).

***Cell migration*** – Cells were grown to confluency in 24 well-chambers and serum-deprived for 24 hours. For the experiments with toxin B cells (TcdB) were preincubated for 24 hours with 10 (DU 145) or 100 pg/ml (PC-3) TcdB follwed by scratching the monolayer and stimulation of the cells. To scratch the monolayer in a standardized fashion, a mechanotransductor was used as previously described [18]. The wells were marked across the wounded area to ensure documentation of the same region. Cells were washed 3 times with PBS and the wells were refilled with medium containing 1 mM hydroxy urea (Sigma-Aldrich) to inhibit cell division. Photos were taken at 0 and 24 hours using an Axiovert S100 microscope (Carl-Zeiss). The area invaded by tumor cells was calculated using the AxioVision Software V4.6.0 (Carl-Zeiss Imaging Solutions).

***DNA synthesis*** - For the measurement of mitogenesis, PC-3 cells were seeded in serum-free medium in 24-well culture plates at a density of 2 x 10^6^ cells/ml, incubated for 24 h with the indicated agonists at 37°C, and pulsed with [^3^H]thymidine (5 µCi/ml) for the last 4 h. After removing of the medium, cells were washed 2 times with ice-cold Hank’s balanced salt solution and treated with 10% trichloroacetic acid. After centrifugation, the pellet was resuspended in 0.5 M NaOH and the incorporated radioactivity was determined by liquid scintillation counting. Results are expressed as percentage of [^3^H]thymidine incorporation in comparison to control.

***Affinity precipitation of GTP-bound RhoA and Rap1*** *–* Affinity precipitation of activated Rap1 and RhoA was performed as described [10, ]. Briefly, 3x 10^6^ PC-3 cells were serum-deprived for 24 hours, stimulated for the indicated periods of time with the indicated agents and lysed. Following this, lysates were cleared by centrifugation and activated Rap1 or RhoA was extracted with glutathione *S*-transferase (GST)-tagged Ral RBD (Rap-binding domain of the Ral guanine nucleotide dissociation stimulator) or Rhotekin, bound to glutathione Sepharose beads. Immunoblotting was performed with an anti-Rap1 (Santa Cruz, 121, sc-65) or anti-RhoA antibody (Cytoskeleton, #ARH01).

***Immunofluorescence microscopy*** – Cells were grown on coverslips, serum-deprived for 24 hours followed by stimulation with the indicated agents for 1 hour. Cells were fixed for 15 min in freshly prepared 3% paraformaldehyde, permeablized (0,05% Triton X-100) and stained for filamentous actin (F-actin) by incubation with AlexaFluor 488-conjugated phalloidin for 15 min according to the manufacturers protocol. Finally, slides were mounted with Aquatex (Merck KGaA) and morphologic changes were visualized with a fluorescence microscope (Axiovert S 100, Zeiss).

***Flow cytometry -*** Cells were trypsinized, stained with AlexaFluor 488-conjugated phalloidin, and analyzed by flow cytometry as described [13]. Cells were gated according to their scatter properties and the mean fluorescence of Alexa flour 488 was determined (Cytomics FC 500, Beckmann Coulter).

***Data presentation and statistical analysis*** – Data are means ± SEM of *n* independent experiments. Statistical significance was determined by one-way ANOVA followed by the Bonferroni test for multiple comparisons. P < 0.05 was considered significant.
